# Pre-radiotherapy systemic immune inflammation index associated with overall survival in patients with advanced EGFR mutant non-small cell lung cancer receiving thoracic radiotherapy

**DOI:** 10.1007/s12094-022-02936-2

**Published:** 2022-09-07

**Authors:** Dujuan Chen, Hongyue Qin, Guangchuan Deng, Qi Wang, Haiyong Wang, Xijun Liu

**Affiliations:** 1 Dongming People’s Hospital, Heze, Shandong Province China; 2 Shandong First Medical University and Shandong Academy of Medical Sciences, Jinan, 250117 Shandong Province China; 3 Department of Radiation Oncology, Shandong First Medical University and Shandong Academy of Medical Sciences, Shandong Cancer Hospital and Institute, Jinan, 250117 Shandong Province China; 4 Department of Internal Medicine-Oncology, Department of Radiation Oncology, Shandong First Medical University and Shandong Academy of Medical Sciences, Shandong Cancer Hospital and Institute, Jinan, 250117 Shandong Province China

**Keywords:** Systemic immune-inflammation index, Thoracic radiotherapy, Non-small-cell lung cancer, Epidermal growth factor receptor

## Abstract

**Purpose:**

This study aimed to investigate the prognostic potential of the pre-radiotherapy systemic immune-inflammation index (SII) for the survival of advanced lung adenocarcinoma patients with epidermal growth factor receptor (*EGFR*) mutations, which might provide a basis for optimizing the comprehensive treatment scheme.

**Methods:**

A total of 111 lung adenocarcinoma patients with *EGFR mutations*, who received thoracic radiotherapy, were included in this retrospective study. The primary endpoint of the study was based on the overall survival (OS) of patients. The receiver operating characteristic (ROC) curve analysis was performed to determine the optimal cut-off value of each immune inflammation index. Kaplan–Meier analysis was performed for the comparison of OS. The Cox proportional-hazard model was used for the multivariate and univariate regression analyses to determine the correlations of prognostic factors with the disease.

**Results:**

SII was divided into the high SII group (≥ 620.2; 45.95%) and the low SII group (SII < 620.2; 54.05%) based on the optimal cutoff values. The median OS rates were 53.3 and 33.3 months in the low and high SII groups, respectively, showing statistically significant differences ( hazard ratio (HR) = 0.459; 95% CI 0.286–0.736; *P* < 0.001). The multivariate analysis showed that, after adjusting for the significant covariates, the SII values were independently associated with the improved OS of the patients (adjusted HR = 0.444; 95% CI 0.279–0.709; *P* = 0.001). The low NLR values were associated with the better OS of patients (HR = 0.509; 95% CI 0.326–0.792;* P* = 0.005) and vice versa (HR = 0.422; 95% CI 0.213–0.836; *P* < 0.001). The patients in the low LMR group before radiotherapy exhibited longer OS as compared to those in the high LMR group (HR = 0.497; 95% CI 0.308–0.802; *P* = 0.001).

**Conclusions:**

This study showed that these inflammatory indices might have an important prognostic potential for advanced lung adenocarcinoma patients with *EGFR* mutations, receiving thoracic radiotherapy and might provide a basis for the individualized treatment of these patients.

## Introduction

Molecular targeted therapy has made great progress in the treatment of advanced epidermal growth factor receptor (EGFR) mutant non-small cell lung cancer (NSCLC) [1]. At present, EGFR tyrosine kinase inhibitors have become the first-line treatment for patients with EGFR-sensitive mutation in stage IV NSCLCs. However, almost all the patients experienced drug resistance after 8–12 months of progression-free survival (PFS) receiving first-line EGFR TKIs [2, 3]. From the perspective of progression sites after receiving EGFR TKIs treatment, a study showed that more than 1/3 of the patients progressed in or near the primary lesions. Therefore, it is particularly important to timely perform thoracic radiotherapy in the management of disease during whole course of patients [4]. A study showed that curative radiotherapy for primary lung lesion can prolong survival in patients with lung adenocarcinoma following EGFR-TKI treatment, involving both patients with oligometastasis and polymetastasis [5]. Some clinical studies have shown that EGFR TKIs combined with thoracic radiotherapy for advanced NSCLCs with EGFR mutation provides long-term control of primary lung tumors [6–8]. Our previous study also confirmed that thoracic radiotherapy was associated with prolonged median OS for this subpopulation patients [9].

However, there is still no mature biomarker for predicting the efficacy of thoracic radiotherapy for this subgroup of patients. Inflammation plays a fundamental role in the tumorigenesis and progression of patients with advanced non-small cell lung cancer [10, 11]. The mediators and cellular effectors of inflammation are important constituents of the local microenvironment of tumors. Regardless of its origin, ‘smouldering’ inflammation in the tumor microenvironment has many tumor-promoting effects. It contributes to the proliferation and survival of malignant cells, promotes angiogenesis and metastasis, destroys the adaptive immune response, and changes the response to radiotherapy. Among those inflammatory indices, neutrophil, platelet and lymphocyte count are all related to the prognosis of lung cancer patients. Otherwise, SII is a novel immune inflammation-based prognostic score that includes these indexes. It was reported that high systemic immune inflammation index (SII) value was related to poor prognosis in a variety of cancers. Some studies showed that SII can be used to predict the survival of patients with Intrahepatic Cholangiocarcinoma (ICCA)、pulmonary sarcomatoid carcinoma (PSC)、resectable gastroesophageal adenocarcinomas and anal cancer [12–15]. The results showed that the overall survival of the high SII group was significantly more worsen than that of the low SII group. There are few studies on the relationship between SII and radiotherapy efficacy. Pre-clinical models reported that combining targeted radiation therapy with a neutrophil stimulant may enhance anti-tumor immunity, causing neutrophil-mediated tumor cell death [16].

The prognostic value of SII in patients with EGFR mutant lung adenocarcinoma receiving thoracic radiotherapy is not clear. Based on our previous study, the main purpose in this retrospective study was to investigate the prognostic value of SII and related inflammatory factors (NLR, PLR and LMR) before thoracic radiotherapy for predicting OS in patients with advanced EGFR mutant lung adenocarcinomas.

## Materials and methods

### Study population and data collection

In this retrospective study, advanced lung adenocarcinoma patients with *EGFR* mutations were recruited from 2008 to 2019. 111 patients of advanced lung adenocarcinoma patients with *EGFR* mutations, who were treated in Shandong cancer hospital, were screened from September 2008 to April 2019. The criteria for the inclusion of patients in this study were as follows: (1) the patients were pathologically diagnosed with primary lung adenocarcinoma; (2) the patients had mutations in *EGFR* exons 18, 19, 20, or 21; (3) the disease stage was determined using computed tomography (CT), magnetic resonance imaging (MRI), positron emission tomography CT (PET-CT), or invasive examination (aspiration cytology); (4) the detailed basic and treatment information of the patients was available; (5) the patients received EGFR-TKIs treatment (gefitinib, erlotinib, icotinib, and afatinib) and thoracic radiotherapy. On the other hand, the patients with second diseases, such as essential thrombocythemia, chronic myeloid leukemia (CML), chronic inflammatory diseases, and autoimmune diseases, were excluded.

Demographic and clinical data were retrieved from the medical record database. The basic information of patients was recorded based on the hospitalization at their first admission. The treatment information was recorded by the attending doctors. All the data, including age, gender, smoking history, type of gene mutations, tumor node metastasis (TNM) stage, pathological characteristics, and treatment information, were extracted according to the unified requirements. The staging of NSCLC was identified based on the TNM Staging Manual of the 8th edition of the American Joint Commission on Cancer (AJCC). The pathological types were analyzed and identified by pathological experts.

### Collection of blood samples and calculation of blood cell count

Venous blood was collected one week before thoracic radiotherapy and the required inflammatory markers and immune cell counts were identified. The N, L, monocytes (M), and P counts were obtained from the complete blood count (CBC), which was performed for each patient before thoracic radiotherapy. Finally, based on these cell counts, four indices were calculated as follows: SII was calculated as P × N/L; NLR was calculated as N/L; PLR was calculated as P/L; and LMR was calculated as L/M.

### Treatment and follow-up

All the patients received EGFR-TKIs (gefitinib, erlotinib, icotinib, and afatinib) as first-line or second-line treatments. They also received other systemic treatments, such as chemotherapy, anti-angiogenic drugs, etc. In addition, the patient received thoracic or other organs radiotherapies, including the brain, bone, adrenal gland, liver, and spleen. Radiotherapy for the primary lung tumors and thoracic metastatic lymph nodes was classified as thoracic radiotherapy.

All the patients were examined and followed up regularly after treatment. The curative effects were mainly evaluated using thoracic CT, brain MRI, and bone scanning. PET-CT was performed based on the patient's conditions or needs. The patients were followed up every three months for two years, then every six months for three years, and then once a year. The follow-up information was obtained by contacting the patients using the telephone or consulting the clinical database. The primary endpoint of the study was the OS of the patients. OS referred to the time from disease diagnosis to death (from any cause).

### Statistical analyses

The data analysis was performed using SPSS. Descriptive analysis was used for all the variables. Categorical variables were expressed as frequencies and percentages, while the continuous variables were expressed as medians. The receiver operating characteristic (ROC) curve analysis was performed to determine the optimal cut-off value of each immune-inflammation index. The Kaplan–Meier method and chi-square (χ^2^) test were performed for the comparison of OS and categorical variables, respectively. The Cox proportional-hazard model was used for the multivariate and univariate regression analyses to determine the prognostic factors related to the disease. The hazard ratios (HRs) were reported as the relative risks with corresponding 95% confidence intervals (CIs). A *P* value of < 0.05 was considered statistically significant.

## Results

### Patient characteristics

The study included a total of 111 patients, who received thoracic radiotherapy between September 2008 to April 2019. The majority of the patients were females (71, 63.96%) and non-smokers (90, 81.08%). Among the 111 patients, 80 patients (72.07%) died and 31 patients (27.93%) survived at the endpoint of the study. The median age of all the patients was 53 years, ranging from 32 to 82 years. A total of 48 (43.24%) and 51 (45.95%) patients had mutations in the exons 19 and 21 of *EGFR*, respectively. Radiotherapy was performed at different sites, including thorax (111, 100%), brain (59, 53.15%), bone (49, 44.14%), liver (3, 2.70%), adrenal gland (3, 2.70%), and other organs (4, 3.60%) and 89 patients (80.18%) received radiotherapies at the metastatic sites. The clinicopathological characteristics of the patients are summarized in Table 1.

**Table 1:** Characteristics of 111 advanced lung adenocarcinoma patients

Characteristic	Total (*N* = 111)	%
Gender		
Male	40	36.04%
Female	71	63.96%
Age		
Median age, year (range)52.5		
≥ 60	33	29.73%
< 60	78	72.27%
Tobacco smoking		
Yes	21	18.92%
No	90	81.08%
EGFR mutation		
19	51	45.95%
Other	12	10.81%
21	48	43.24%
NLR		
≥ 2.32	73	65.77%
< 2.32	38	34.23%
PLR		
≥ 251.5	23	20.72%
< 251.5	88	79.28%
LMR		
≥ 2.76	66	59.46%
< 2.76	45	40.54%
SII		
SII ≥ 620.2	51	45.95%
SII < 620.2	60	54.05%
Metastatic site		
Brain	68	61.26%
Bone	78	70.27%
Liver	3	2.70%
Adrenal gland	3	2.70%
Other	4	3.60%
Radiotherapy site		
Brain	59	53.15%
Bone	49	44.14%
Liver	3	2.70%
Adrenal	3	2.70%
Other	4	3.60%
Number of radiotherapy		
1	25	22.52%
2	53	47.75%
3	26	23.42%
4	3	2.70%
5	3	2.70%
6	1	0.90%
Number of radiotherapy in metastatic sites		
0	22	19.82%
1	57	51.35%
2	29	26.13%
3	3	2.70%

*EGFR* epidermal growth factor receptor, *TKIs* tyrosine kinase inhibitors

### ROC curve

**Fig. 1:**
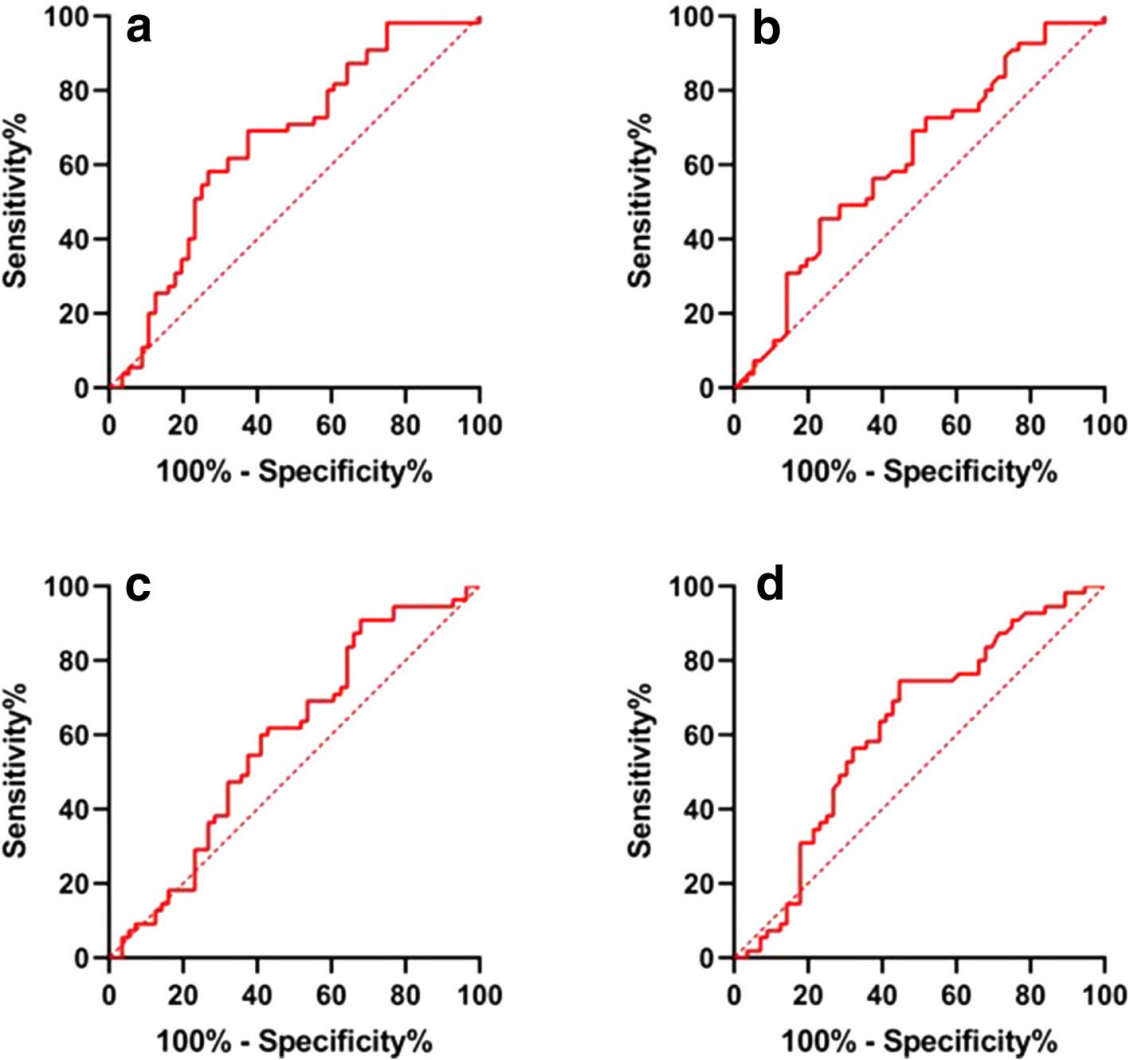
ROC curve of SII (**a**) NLR (**b**) PLR (**c**) LMR (**d**)

The optimal cutoff value for SII was 620.2 with the area under the curve (AUC) of 0.659 (sensitivity: 69.09%, 95% CI 55.97–79.72%; specificity: 62.50%, 95% CI 49.41–73.99%) (Fig. 1a). SII was divided into high SII group (SII ≥ 620.2; 51; 45.95%) and low SII group (SII < 620.2; 60; 54.05%) based on the optimal cutoff values. The optimal cutoff value for NLR was 2.32 with the AUC of 0.614 (sensitivity: 45.45%, 95% CI: 33.03–58.48%; specificity: 76.79%, 95% CI 64.23–85.90%), respectively (Fig. 1b). NLR was divided into high NLR group (NLR ≥ 2.32; 73; 65.77%) and low NLR group (NLR < 2.32; 38; 34.23%) based on the optimal cutoff values. The optimal cutoff value of PLR was 251.5 with an AUC of 0.586 (sensitivity: 90.91%, 95% CI 80.42–96.05%; specificity: 32.14%, 95% CI 21.40–45.18%) (Fig. 1c). Based on the optimal cutoff values of PLR, the patients were divided into two groups, including low PLR group (PLR ≤ 251.5, 88 patients) and high PLR group (PLR ˃251.5, 23 patients). The optimal cutoff value of LMR was 2.76 with an AUC of 0.620 (sensitivity: 74.55%, 95% CI 61.70–84.19%; specificity: 55.36%, 95% CI 42.41–67.61%) (Fig. 1d). Based on the optimal cutoff values of LMR, the patients were divided into two groups, including low LMR group (LMR ≤ 2.76, 45 patients) and high LMR group (LMR ˃2.76, 66 patients).

### Survival status of the entire study cohort

**Fig. 2:**
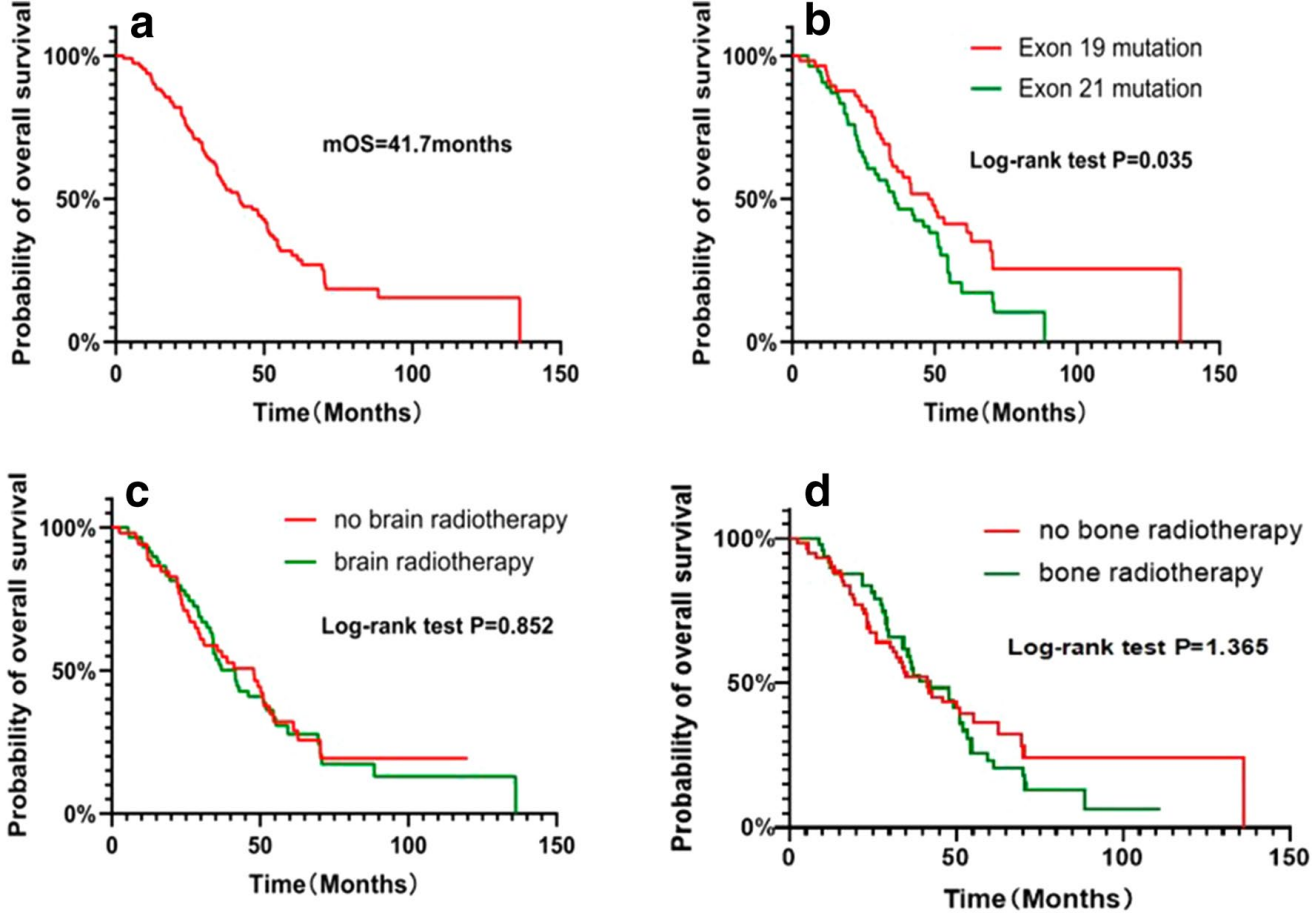
Overall survival (OS) of the entire cohort (**a**) and of patients stratified according to EGFR mutation status (**b**). Effect of brain radiotherapy on overall survival (OS) in patients with brain metastasis (**c**). Effect of bone radiotherapy on overall survival (OS) in patients with bone metastasis (**d**)

The duration from the beginning of diagnosis to death from any cause or the last follow-up was defined as the OS of the patients. The patients, who were lost to follow-up or survived until the end of this study's observation time, were defined as censored. After the follow-up duration, a total of 80 patients (72.07%) died in the entire cohort. The median OS (mOS) of the patients was 41.7 months. The 1-, 3-, and 5-year survival rates of the entire cohort were 91.89%, 55.11%, and 28.64%, respectively (Fig. 2a). There were significant differences in the OS rates of the patients with mutations in *EGFR* exon 19 and those with mutations in *EGFR* exon 21 (HR = 0.628; 95% CI 0.401–0.982; log-rank *P* = 0.035) (Fig. 2b). There was no significant difference in the OS rates of the 59 patients, who received brain radiotherapy as compared to those who did not receive brain radiotherapy (HR = 0.959; 95% CI 0.618–1.488; log-rank *P* = 0.852) (Fig. 2c). Similarly, among the 78 patients with bone metastases, there was no significant difference in the OS rates of the 49 patients who received bone radiotherapy as compared to those who did not receive bone radiotherapy (HR = 0.879; 95% CI 0.566–1.365; log-rank *P* = 1.365;) (Fig. 2d).

### Effects of SII, NLR, PLR, and LMR on the patients’ OS

**Fig. 3:**
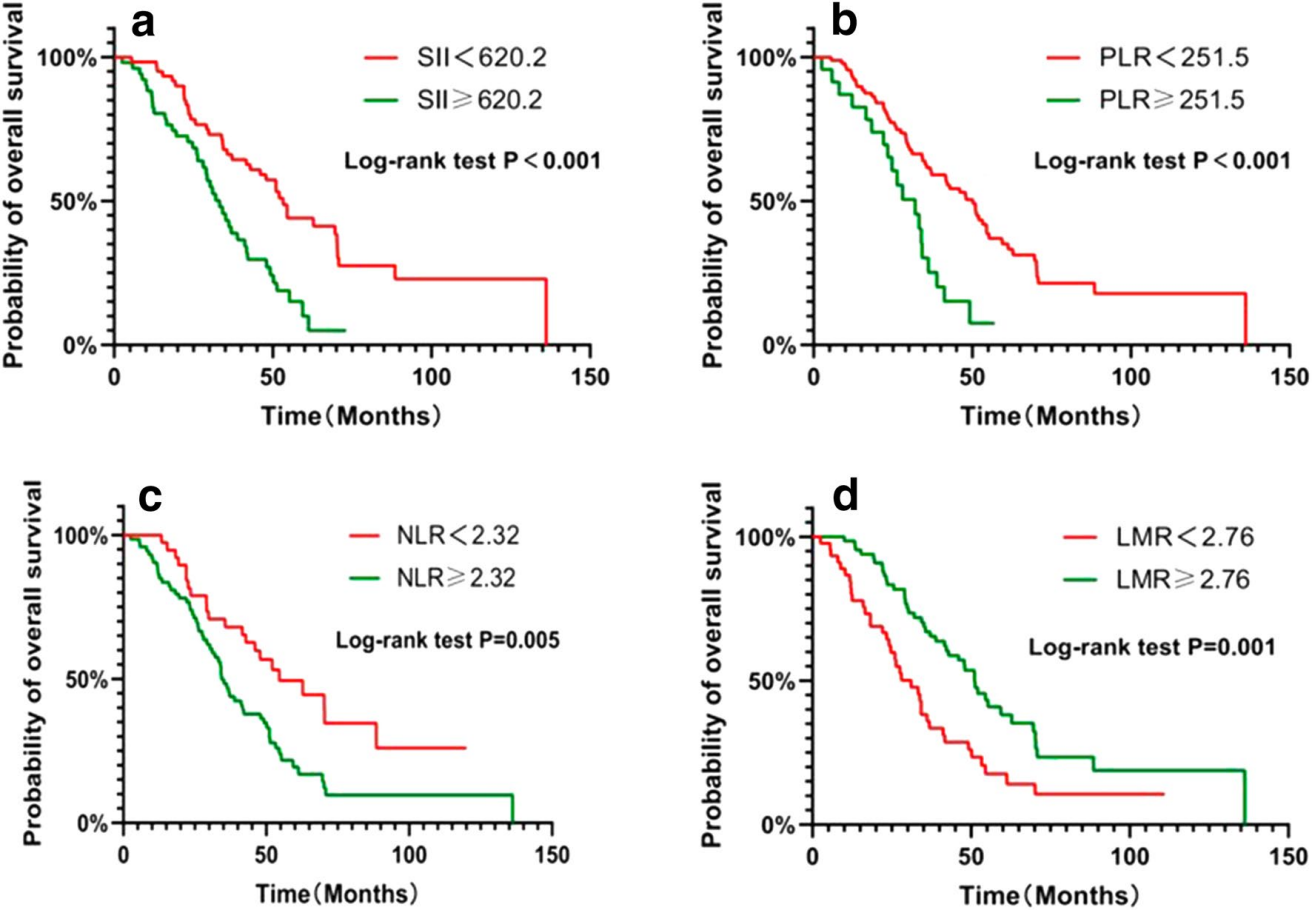
Comparison of OS between high SII groups and low SII groups (**a**). Comparison of OS between high NLR groups and low NLR groups (**b**). Comparison of OS between high PLR groups and low PLR groups (**c**). Comparison of OS between high LMR groups and low LMR groups (**d**)

The patients in the low NLR group were associated with better OS (HR = 0.509; 95% CI 0.326–0.792; *P* = 0.005) (Fig. 3b). The patients in the high PLR group showed significantly poor OS (HR = 0.422; 95% CI 0.213–0.836; *P* < 0.001) (Fig. 3c). The patients in the low LMR group before radiotherapy showed longer OS as compared to those in the high LMR group (HR = 0.497; 95% CI 0.308–0.802; *P* = 0.001). The mOS of the patients in the low SII group (53.3 months) was better as compared to that in the high SII group (33.3 months) (Fig. 3d). This difference in the OS of the patients in the low and high SII groups was statistically significant (HR = 0.459; 95% CI 0.286–0.736; *P* < 0.001) (Fig. 3a).

### Univariate and multivariate regression analyses of the independent predictors of OS

*χ*^2^ test was performed to analyze the differences in the clinicopathological factors of patients in the different SII groups to eliminate the effects of other clinicopathological factors on the OS of patients. There were no differences in age, sex, *EGFR* mutation type, smoking status, radiotherapy site, or the number of radiotherapies in metastatic sites in the two groups (Table 2). The univariate and multivariate regression analyses of OS were performed using the Cox regression model based on the ages, genders, smoking statuses, type of mutations in *EGFR* gene, NLR, PLR, LMR, and SII values. The univariate regression analysis revealed the types of mutations in the *EGFR* gene, NLR, PLR, LMR, and SII showed significant potential as prognostic factors for predicting the OS of the patient. The multivariate regression analysis showed that, after adjusting for the significant covariates, including age, gender, smoking status, type of mutations in *EGFR* gene and SII values, the SII value was independently associated with the improved OS of patients (adjusted HR = 0.444; 95% CI 0.279–0.709; *P* = 0.001) (Table 3).

**Table 2:** Characteristics of patients in the two groups and with the *χ*^2^ test for categorical variables

	All patients (*n* = 111)	SII ≥ 620.2	SII < 620.2	*P* value
Gender				0.370
Male	40 (36.04%)	21	19	
Female	71 (63.96%)	31	40	
Age				
Median age, year (range) 53				0.919
≥ 60	33 (29.73%)	16	17	
< 60	78 (72.27%)	37	41	
Tobacco smoking				0.294
Yes	21 (18.92%)	12	9	
No	90 (81.08%)	40	50	
EGFR mutation				0.927
19	51 (45.95%)	24	27	
Orthers	12 (10.81%)	5	7	
21	48 (43.24%)	23	25	
NLR				< 0.001
≥ 2.32	73 (65.77%)	50	23	
< 2.32	38 (34.23%)	2	36	
PLR				< 0.001
≥ 251.5	23 (20.72%)	19	4	
< 251.5	88 (79.28%)	33	55	
LMR				< 0.001
≥ 2.76	66 (59.46%)	21	45	
< 2.76	45 (40.54%)	31	14	
Radiotherapy site				
(Brain) yes	59 (53.15%)	31	28	0.200
No	52 (46.85%)	21	31	
(Bone) yes	49 (44.14%)	23	26	0.986
No	62 (55.86%)	29	33	
Number of radiotherapy				
1, 2, 3	10 4(93.69%)	48	56	0.573
4, 5, 6	7 (6.31%)	4	3	
Number of radiotherapy in metastatic sites				
Yes	89 (80.18%)	43	46	0.533
No	22 (19.82%)	9	13

**Table 3:** Univariable and Multivariable analyses of covariable associated with OS

Clinical basic statistics for 121 patients	Univariate analysis		Multivariate analysis	
	HR (95% CI)	*P* value	HR (95% CI)	*P* value
Gender	0.892 (0.568–1.401)	0.622	0.681 (0.346–1.340)	0.266
Male				
Female				
Age	0.723 (0.447–1.169)	0.725	1.172 (0.712–1.929)	0.532
Median age, year (range)53				
≥ 60				
< 60				
Tobacco smoking	1.416 (0.836–2.39)	0.090	0.485 (0.218–1.078)	0.076
Yes				
No				
EGFR mutation	0.628 (0.401–0.982)	0.035	0.711 (0.446–1.134)	0.153
19				
21				
NLR	0.509 (0.326–0.792)	0.005		
≥ 2.32				
< 2.32				
PLR	0.422 (0.213–0.836)	< 0.001		
≥ 251.5				
< 251.5				
LMR	0.497 (0.308–0.802)	0.001		
≥ 2.76				
< 2.76				
SII	0.459 (0.286–0.736)	< 0.001	0.444 (0.279–0.709)	0.001
≥ 620.2				
< 620.2				
Radiotherapy site				
Brain (yes vs no)	0.959 (0.618–1.488)	0.852		
Bone (yes vs no)	0.879 (0.566–1.365)	0.562		
Number of radiotherapy				
1, 2, 3	0.808 (0.324–2.013)	0.612		
4, 5, 6				

## Discussion

This study showed that the mOS of all patients who received thoracic radiotherapy was 36.9 months. The OS of the patients was significantly prolonged as compared to those reported in previous studies, indicating that thoracic radiotherapy could significantly improve the prognosis of lung adenocarcinoma patients with *EGFR* mutation [9]. As a local treatment, thoracic radiotherapy could improve the disease conditions of advanced lung adenocarcinoma patients with *EGFR* mutation [17]. The timely administration of thoracic radiotherapy can significantly improve the survival rate of advanced lung cancer patients [4]. On the other hand, some patients achieved shorter overall survival after receiving thoracic radiotherapy. Therefore, the patients, who do not benefit from thoracic radiotherapy in this circumstance, should be excluded based on biomarkers, thereby avoiding the side effects of radiotherapy on normal lung function.

In this study, several inflammatory indices, including NLR, PLR, LMR, and SII, were selected to evaluate their prognostic potential [18]. The results showed that these indices were all significantly correlated with the prognosis of the NSCLC patients with metastatic *EGFR* mutation. The optimal prognostic potential of these indices was determined based on the ROC curve, and the patients were divided into high- and low-value groups. Kaplan–Meier curves showed that the patients in the low-value group had longer OS as compared to those in the high-value group. SII is a comprehensive index, correlated with other indices. Therefore, only SII was included in the multivariate analysis. Both the univariate and multivariate regression analyses showed that SII was a significant independent prognostic factor for the OS of patients. Previous studies showed that the response rates of the patients in the low SII groups to radiotherapy were significantly better as compared to those in the high SII groups of the stage III NSCLC patients [19], 19, the SCLC patients [21], the early stage NSCLC [22], the NSCLC patients with brain metastases [23] which were consistent to the results in our study.

Although the cutoff value of SII was not consistent with those of the previous studies, the results showed that the SII value was significantly negatively correlated with the OS of the patients, which was consistent with the results in the current study. Previous studies had mainly focused on the prognostic potential of the inflammatory indices in early stage or locally advanced NSCLC patients, receiving radical thoracic radiotherapy. There are few reports on the prognostic role of this indicator in advanced non-small cell lung cancer. In this study, the study population was expanded to the stage IV NSCLC patients, who received local thoracic radiotherapy. This showed that the pre-radiotherapy SII, NLR, PLR, and LMR levels were the key prognostic factors of metastatic NSCLC with *EGFR* mutations; the low NLR, PLR, and LMR levels were associated with a better prognosis for patients.

These inflammatory indices were selected for analysis in the current study due to the following reasons. First, since Virchow identified the correlation between cancer and inflammation in the nineteenth century, numerous studies have suggested that inflammatory markers play a vital role in tumor progression and metastasis [24, 25]. Second, radiotherapy mainly activates the immune system by enhancing antigen presentation and increasing immune cell infiltration [26]. It can increase the lymphocyte count [27]. Cancer immunotherapy can increase sensitivity to radiation treatment by activating T-cells [28]. Radiotherapy can also increase the neutrophils count, which can reduce the sensitivity of radiotherapy and can suppress the T-cell activation to promote immune evasion [29, 30]. The increase of neutrophils will promote the release of inflammatory factors such as vascular epithelial growth factor (VEGF), interleukin-8 (IL-8), interleukin-16 and the production of a series of proteases [31]. In addition, these inflammatory cytokines produced in the tumor microenvironment promote the development and progression of tumors. Lymphopenia reversely causes neutrophilia, which indirectly reduces sensitivity to radiotherapy. lymphopenia may refect a lower number of CD4 + T helper lymphocytes, resulting in a poorer lymphocyte-mediated immune response to malignancies [32]. Platelets keep circulating tumor cells (CTCs) from shearing stresses in vessels and inducing CTC epithelial-mesenchymal transition [33]. Third, the final prognosis could easily be predicted, providing a widely applicable prognostic tool for clinical practices. The SII, as a comprehensive index, reflects the combination of nonspecific inflammation and immune function. An increase in the SII value usually indicates thrombocytopenia, neutropenia, or lymphocytopenia, showing an increase in an inflammatory response and immune dysfunction. Previous studies showed that the unbalanced inflammatory state or defects in the immune response could trigger the proliferation, invasion, and metastasis of tumors [34]. As previously reported, this can be explained by the inherent functions of the three types of blood cells.

There were certain limitations to this study, which could not be ignored. This was a retrospective single-center-based study with a relatively small number of patients. Therefore, the sample size in the subgroup analyses might not be sufficient to be used as a representative. Hence, future studies should register more advanced NSCLC patients treated with radiotherapy and should focus on multicenter collaborative prospective studies.

## Conclusions

This study showed that these inflammatory indices might have an important prognostic potential for advanced lung adenocarcinoma patients with *EGFR* mutations, receiving thoracic radiotherapy and might provide a basis for the individualized treatment of these patients.

## Data Availability

The datasets generated for this study are available on request to the corresponding author.

## Abbreviations

NSCLC: Non-small-cell lung cancer
OS: Overall survival
SII: Systemic immune-inflammation index
TRT: Thoracic radiotherapy
EGFR: Epidermal growth factor receptor

## References

[1] da Cunha SG, Shepherd FA, Tsao MS (2011). EGFR mutations and lung cancer. Annu Rev Pathol.

[2] Gelatti ACZ, Drilon A, Santini FC (2019). Optimizing the sequencing of tyrosine kinase inhibitors (TKIs) in epidermal growth factor receptor (EGFR) mutation-positive non-small cell lung cancer (NSCLC). Lung Cancer.

[3] Wu SG, Shih JY (2018). Management of acquired resistance to EGFR TKI-targeted therapy in advanced non-small cell lung cancer. Mol Cancer.

[4] Tang Y, Xia B, Xie R, Xu X, Zhang M, Wu K (2020). Timing in combination with radiotherapy and patterns of disease progression in non-small cell lung cancer treated with EGFR-TKI. Lung Cancer.

[5] Hsu KH, Huang JW, Tseng JS, Chen KW, Weng YC, Yu SL (2021). Primary tumor radiotherapy during EGFR-TKI disease control improves survival of treatment naive advanced EGFR-mutant lung adenocarcinoma patients. Onco Targets Ther.

[6] Wei H, Zhou X, Yang H, Gong Y, Wang J, Xu Y (2021). Stereotactic body radiotherapy to the primary lung lesion improves the survival of the selected patients with non-oligometastatic NSCLC harboring EGFR activating mutation with first-line EGFR-TKIs: a real-world study. J Cancer Res Clin Oncol.

[7] Wang X, Zeng Z, Cai J, Xu P, Liang P, Luo Y (2021). Efficacy and acquired resistance for EGFR-TKI plus thoracic SBRT in patients with advanced EGFR-mutant non-small-cell lung cancer: a propensity-matched retrospective study. BMC Cancer.

[8] Wang X, Lu Z, Zeng Z, Cai J, Xu P, Liu A (2021). Thoracic stereotactic body radiation therapy plus first-line tyrosine kinase inhibitors for patients with epidermal growth factor receptor-mutant polymetastatic non-small-cell lung cancer: a propensity-matched retrospective study. Medicine (Baltimore).

[9] Zhang Y, Wang W, Xu X, Li Y, Zhang H, Li J (2021). Impact of radiotherapy pattern on the prognosis of stage IV lung adenocarcinomas harboring EGFR mutations. Cancer Manag Res.

[10] Budisan L, Zanoaga O, Braicu C, Pirlog R, Covaliu B, Esanu V (2021). Links between infections, lung cancer, and the immune system. Int J Mol Sci.

[11] Ju Q, Huang T, Zhang Y, Wu L, Geng J, Mu X (2021). Systemic immune-inflammation index predicts prognosis in patients with different EGFR-mutant lung adenocarcinoma. Medicine (Baltimore).

[12] Ren A, Li Z, Cheng P, Zhang X, Deng R, Ma Y (2021). Systemic immune-inflammation index is a prognostic predictor in patients with intrahepatic cholangiocarcinoma undergoing liver transplantation. Mediators Inflamm.

[13] Zeng Q, Li J, Sun N, Xue Q, Gao Y, Zhao J (2021). Preoperative systemic immune-inflammation index predicts survival and recurrence in patients with resected primary pulmonary sarcomatoid carcinoma. Transl Lung Cancer Res.

[14] Hu B, Yang XR, Xu Y, Sun YF, Sun C, Guo W (2014). Systemic immune-inflammation index predicts prognosis of patients after curative resection for hepatocellular carcinoma. Clin Cancer Res.

[15] Jomrich G, Paireder M, Kristo I, Baierl A, Ilhan-Mutlu A, Preusser M (2021). High Systemic Immune-Inflammation Index is an Adverse Prognostic Factor for Patients With Gastroesophageal Adenocarcinoma. Ann Surg.

[16] Schernberg A, Blanchard P, Chargari C, Deutsch E (2017). Neutrophils, a candidate biomarker and target for radiation therapy?. Acta Oncol.

[17] Yen YC, Hsu HL, Chang JH, Lin WC, Chang YC, Chang CL (2018). Efficacy of thoracic radiotherapy in patients with stage IIIB-IV epidermal growth factor receptor-mutant lung adenocarcinomas who received and responded to tyrosine kinase inhibitor treatment. Radiother Oncol.

[18] Yucel S, Bilgin B (2021). The prognostic values of systemic immune-inflammation index and derived neutrophil-lymphocyte ratio in EGFR-mutant advanced non-small cell lung cancer. J Oncol Pharm Pract.

[19] Liu J, Li S, Zhang S, Liu Y, Ma L, Zhu J (2019). Systemic immune-inflammation index, neutrophil-to-lymphocyte ratio, platelet-to-lymphocyte ratio can predict clinical outcomes in patients with metastatic non-small-cell lung cancer treated with nivolumab. J Clin Lab Anal.

[20] DelikgozSoykut E, Kemal Y, Karacin C, Karaoglanoglu O, Kurt M, AytacArslan S (2022). Prognostic impact of immune inflammation biomarkers in predicting survival and radiosensitivity in patients with non-small-cell lung cancer treated with chemoradiotherapy. J Med Imaging Radiat Oncol.

[21] Abravan A, Salem A, Price G, Faivre-Finn C, van Herk M (2022). Effect of systemic inflammation biomarkers on overall survival after lung cancer radiotherapy: a single-center large-cohort study. Acta Oncol.

[22] Luo H, Ge H, Cui Y, Zhang J, Fan R, Zheng A (2018). Systemic inflammation biomarkers predict survival in patients of early stage non-small cell lung cancer treated with stereotactic ablative radiotherapy: a single center experience. J Cancer.

[23] Zhang Y, Chen Z, Jin F, Guo D, Chen Q, Liu Z (2021). The value of the systemic immune-inflammation index in predicting survival outcomes in patients with brain metastases of non-small-cell lung cancer treated with stereotactic radiotherapy. Mediators Inflamm.

[24] Diakos CI, Charles KA, McMillan DC, Clarke SJ (2014). Cancer-related inflammation and treatment effectiveness. Lancet Oncol.

[25] Kay J, Thadhani E, Samson L, Engelward B (2019). Inflammation-induced DNA damage, mutations and cancer. DNA Repair (Amst).

[26] Wu M, Liu J, Wu S, Liu J, Wu H, Yu J (2021). Systemic Immune activation and responses of irradiation to different metastatic sites combined with immunotherapy in advanced non-small cell lung cancer. Front Immunol.

[27] Zhang T, Yu H, Ni C, Zhang T, Liu L, Lv Q (2017). Hypofractionated stereotactic radiation therapy activates the peripheral immune response in operable stage I non-small-cell lung cancer. Sci Rep.

[28] Wang Y, Liu ZG, Yuan H, Deng W, Li J, Huang Y (2019). The reciprocity between radiotherapy and cancer immunotherapy. Clin Cancer Res.

[29] Sharabi AB, Lim M, DeWeese TL, Drake CG (2015). Radiation and checkpoint blockade immunotherapy: radiosensitisation and potential mechanisms of synergy. Lancet Oncol.

[30] Coffelt SB, Wellenstein MD, de Visser KE (2016). Neutrophils in cancer: neutral no more. Nat Rev Cancer.

[31] Tong YS, Tan J, Zhou XL, Song YQ, Song YJ (2017). Systemic immune-inflammation index predicting chemoradiation resistance and poor outcome in patients with stage III non-small cell lung cancer. J Transl Med.

[32] Keizman D, Gottfried M, Ish-Shalom M, Maimon N, Peer A, Neumann A (2012). Pretreatment neutrophil-to-lymphocyte ratio in metastatic castration-resistant prostate cancer patients treated with ketoconazole: association with outcome and predictive nomogram. Oncologist.

[33] Stanger BZ, Kahn ML (2013). Platelets and tumor cells: a new form of border control. Cancer Cell.

[34] McLaughlin M, Patin EC, Pedersen M, Wilkins A, Dillon MT, Melcher AA (2020). Inflammatory microenvironment remodelling by tumour cells after radiotherapy. Nat Rev Cancer.

